# FGF22 heterozygous and FGFR2 mosaic mutations in Munro Acne Nevus: a case study^☆^

**DOI:** 10.1016/j.abd.2025.501141

**Published:** 2025-07-14

**Authors:** Jiachen Sun, Yimeng Wang, Lihua Zhang, Chunlei Zhang, Chuan Ma

**Affiliations:** a Department of Dermatology, Peking University Third Hospital, Beijing, China; b Department of Pathology, Fourth Medical Center of Chinese PLA General Hospital, Beijing, China

Dear Editor,

Munro acne nevus, also known as nevus comedonicus (MIM 617025), is a rare developmental anomaly of the pilosebaceous unit, classified as a subtype of epidermal nevus. Recent studies have implicated several genes in its pathogenesis, including FGFR2, FGFR3 and NEK9. Herein, we report a case of Munro acne nevus associated with a germline heterozygous FGF22 mutation (c.104A>G) and a somatic mosaic FGFR2 mutation (c.755G>C).

A 22-year-old female presented with a congenital, asymptomatic plaque on her back that had progressively enlarged (Fig. 1A). Physical examination revealed an irregularly shaped, erythematous patch with well-demarcated borders in the right lumbosacral region. In the affected area dark red to black follicular papules with acuminate tips were observed (Fig. 1B). Comedones and hypopigmented terminal hairs were evident within the affected area. A comprehensive clinical evaluation revealed no systemic abnormalities.

**Figure 1:**
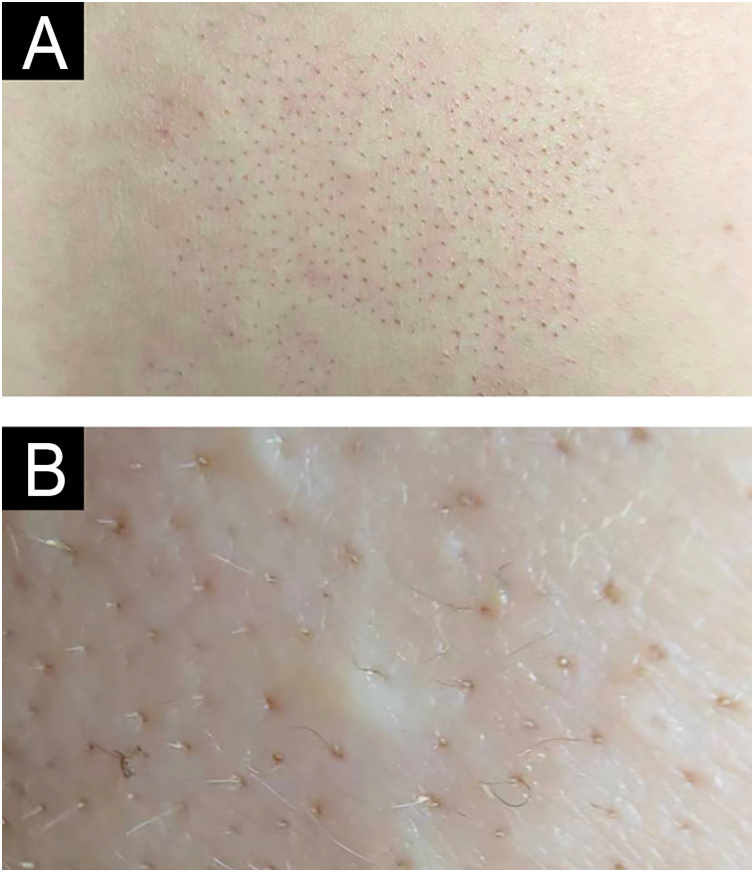
Clinical aspect (A) and close-up view (B).

Histopathology (Fig. 2A) demonstrated mild acanthosis and hyperkeratosis of the epidermis (Fig. 2B), focal basal layer hyperpigmentation, and dermal collagen hyperplasia. A hair follicle with prominent sebaceous hyperplasia was noted (Fig. 2C), accompanied by mild perifollicular fibrosis and lymphocytic infiltrate. The superficial dermis exhibited sparse perivascular lymphocytic infiltration.

**Figure 2:**
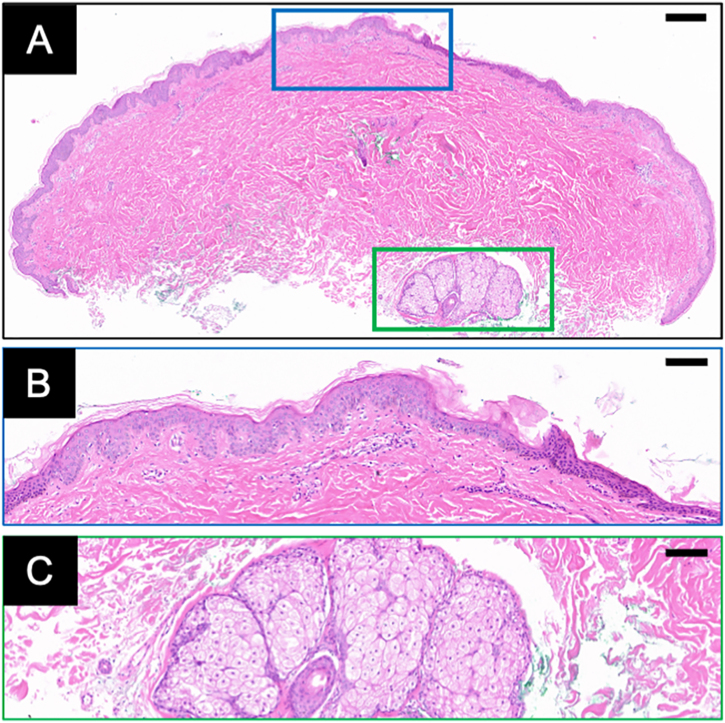
Overview of the lesional biopsy with Hematoxylin & eosin staining (A). The blue and green boxes represent the magnified views of the epidermis (B) and sebaceous glands (C), respectively. Scale bar: 300 μm for (A), 100 μm for (B and C).

Genetic analysis identified a germline heterozygous mutation in *FGF22* (rs574406266, c.104A>G, p.His35Arg) in both lesional skin and peripheral blood, based on Whole Exome Sequencing (WES) of DNA extracted with TIANamp Genomic DNA Kit (DP304, TIANGEN, China). Additionally, a somatic mosaic mutation in *FGFR2* (rs79184941, c.755G>C, p.Ser252Trp) was detected exclusively in the affected skin, with a variant allele frequency of 17%, confirmed by Sanger sequencing (Fig. 3).

**Figure 3:**
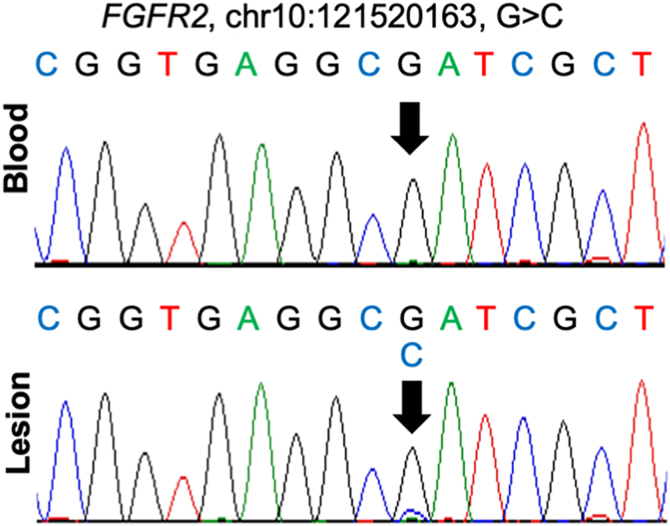
Sanger sequencing validation of the *FGFR2* mutation identified by WES in the patient's peripheral blood and lesional tissue.

Immunofluorescence analysis (Fig. 4A‒B) revealed an uneven distribution of Ki-67-positive cells in the epidermis and sebaceous glands, with a higher abundance compared to normal control skin. Aberrant upregulation of CK6 and downregulation of CK5 were observed in the epidermis and peripheral basal cells surrounding the sebaceous glands. Notably, both basal and suprabasal keratinocytes demonstrated increased and colocalized expression of FGF22 and FGFR2, as evidenced by intense yellow fluorescence signals. In contrast, the sebaceous glands displayed expression of FGFR2, with minimal expression of FGF22.

**Figure 4:**
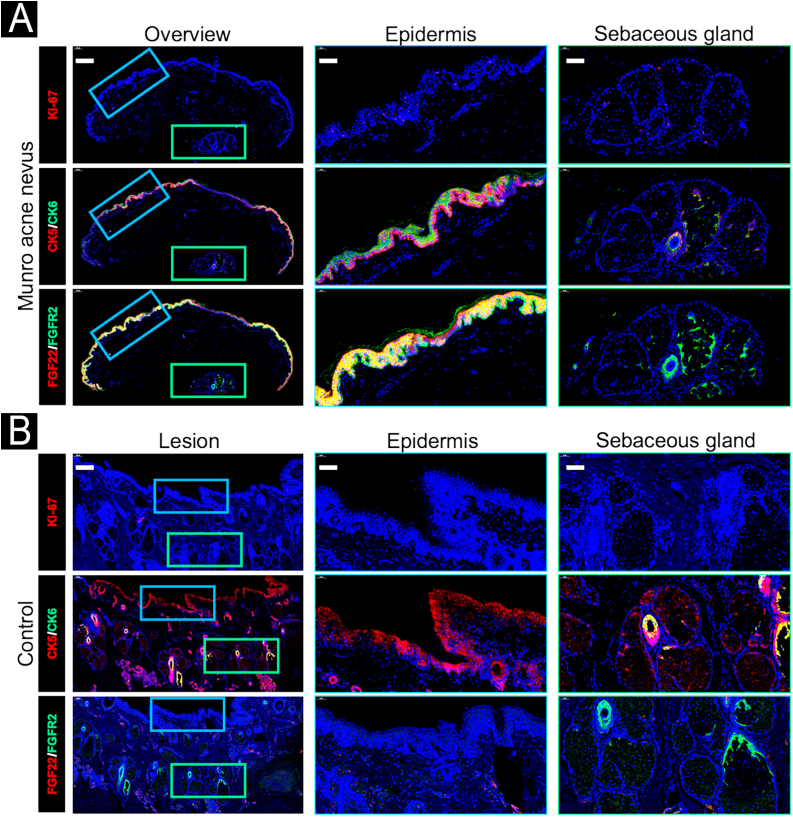
Immunofluorescence staining of the lesional biopsy (A) and healthy control (B). In the lesional skin (A), an uneven distribution of Ki-67-positive cells was observed in the epidermis and sebaceous glands, with higher abundance compared to normal control skin. CK6 was upregulated and CK5 downregulated in the epidermis and peripheral basal cells of the sebaceous glands. Basal and suprabasal keratinocytes showed increased and colocalized expression of FGF22 and FGFR2. In contrast, the sebaceous glands exhibited FGFR2 expression but minimal FGF22. In the healthy control skin (B), there was minimal presence of Ki-67-positive cells or CK6 in the epidermis and sebaceous glands. CK5 was expressed in the epidermis and peripheral basal cells of the sebaceous glands. Basal and suprabasal keratinocytes exhibited no expression of FGF22 or FGFR2. In the peripheral basal cells of the sebaceous glands, FGFR2 expression was observed, with FGF22 absent. Scale bar: 200 μm for overview; 50 μm for the epidermis and sebaceous glands.

Cutaneous mosaic-activating *FGFR2* mutations have been identified in several cases of nevus comedonicus, including c.755G>C (p.Ser252Trp),^1^, ^2^ c.758C>G (p.Pro253Arg),^3^ and c.1144T>C (p.Cys382Arg).^4^ Apert syndrome also involves mutations at these sites of *FGFR2*, but is typically associated with extensive acne, along with craniosynostosis and severe syndactyly of the hands and feet.^5^ The p.Ser252Trp and p.Pro253Arg mutations of *FGFR2* are located in the extracellular topological domain, specifically in the linker region between immunoglobulin-like domains II and III. These gain-of-function mutations create additional non-specific FGF-FGFR contacts, enabling pathological binding of FGFs to FGFR2. Our case firstly reports a Chinese Munro acne nevus patient harboring the *FGFR2* c.755G>C (p.Ser252Trp) mutation. Interestingly, this specific mutation has been associated with diverse phenotypes: M. Larsabal et al.^1^ described naevoid acanthosis nigricans with localized skin thickening and hyperpigmentation, while B.C. Melnik et al.^2^ reported inflammatory papules and pustules with hypopigmentation. In comparison, our case exhibited relatively milder skin lesions compared to these two cases.

FGF22 is a potent activator of FGFR2 in the skin, binding to FGFR2 IgIIIc. *FGF22* p.His35Arg (c.104A>G) mutation is novel in epidermal nevus and remains largely uncharacterized (SIFT score = 0.001, Polyphen2 HVAR score = 0.998). His35 is proximal to the N-terminal signal peptide region, suggesting that p.His35Arg mutation may affect FGF22 secretion and extracellular localization. In our case, FGFR2 and FGF22 were clearly colocalized in the epidermis, suggesting a potential ligand-receptor interaction *in situ*. Along with increased CK6 and Ki-67 expression in this area, this indicates that disrupted epidermal stratification and abnormal keratinocyte proliferation may be associated with FGF22/FGFR2 signaling. We propose a model wherein mutant FGF22 acts as a hyperactive ligand for constitutively activating FGFR2, resulting in sustained activation of downstream pathways.

## Financial support

None declared.

## Author's contribution

Jiachen Sun: Performed the experiments and data analysis, and wrote the manuscript, critically reviewed the manuscript.

Yimeng Wang: Performed the experiments and data analysis, and wrote the manuscript, critically reviewed the manuscript.

Lihua Zhang: Performed the experiments and data analysis, and wrote the manuscript, provided the pathological analysis, critically reviewed the manuscript.

Chunlei Zhang: Conceptualized the study and designed experiments; critically reviewed the manuscript.

Chuan Ma: Conceptualized the study and designed experiments; critically reviewed the manuscript.

## Conflicts of interest

None declared.
